# Supplementary material for: Effects of Dietary Glucose and Fructose on Copper, Iron, and Zinc Metabolism Parameters in Humans
Source: Nutrients. 2020 Aug 25;12(9):2581. doi: 10.3390/nu12092581 (PMC7551875; doi:10.3390/nu12092581)
Supplement: Supplementary file 1 [file nutrients-12-02581-s001.pdf]

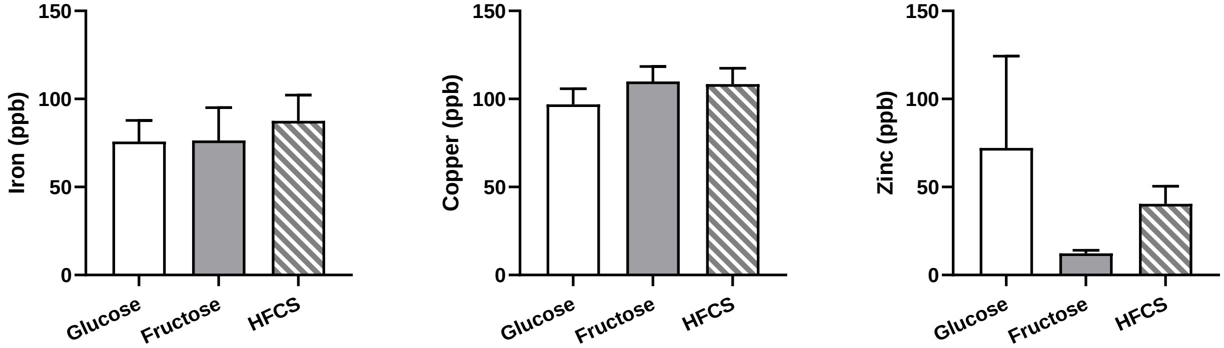

Figure S1. Metal concentrations in glucose-, fructose-, or HFCS-sweetened beverages consumed by subjects (n=6). Mean ± SEM are shown. One-way ANOVA were used to analyze significance between metal levels in sweetened beverages; no significant differences were found.
